# Genetic mapping and phenotypic analysis of *shot^H.3.2^* in *Drosophila melanogaster*

**DOI:** 10.17912/micropub.biology.000418

**Published:** 2021-07-13

**Authors:** Elyse M Talley, Charlie T Watts, Sonia Aboyer, Madeline G Adamson, Harriet AB Akoto, Haley Altemus, Philip J Avella, Rebecca Bailey, Elizabeth R Bell, Katheryn L Bell, Kelsey Breneman, Jessica S Burkhart, Logan J Chanley, Savannah S Cook, Mackenzie T DesLaurier, Timothy R Dorsey, Cassandra J Doyle, Merris E Egloff, Ayoola S Fasawe, Katy K Garcia, Nathaniel P Graves, Tyler K Gray, Evan M Gustafson, Makayla J Hall, Jaden D Hayes, Lindsay J Holic, Brice A Jarvis, Piotr S Klos, Sidney Kritzmire, Lera Kuzovko, Edwyna Lainez, Shamerra McCoy, James C Mierendorf, Nicole A Neri, Caley R Neville, Kelley Osborn, Kaitlyn Parker, Megan E Parks, Kylee Peck, Robyn Pitt, Matthew E Platta, Brianna Powell, Katalina Rodriguez, Clara Ruiz, Mariah N Schaefer, Amanda B Shields, Jasmine B Smiley, Briona Stauffer, Devan Straub, John L Sweeney, Kaitlyn M Termine, Brett Thomas, Sophia D Toth, Taylor R Veile, Kayla S Walker, Paige N Webster, Brian J Woodard, Quentin L Yoder, McKenzie K Young, McKenzie L Zeedyk, Logan N Ziegler, Kayla L Bieser, David P Puthoff, Joyce Stamm, Alysia D Vrailas-Mortimer, Jacob D Kagey, Julie A Merkle

**Affiliations:** 1 University of Evansville, Evansville, IN USA; 2 Illinois State University, Normal, IL USA; 3 Frostburg State University, Frostburg, MD USA; 4 Nevada State College, Henderson, NV USA; 5 University of Detroit Mercy, Detroit, MI USA

## Abstract

Genetic screens are used to identify genes involved in specific biological processes. An EMS mutagenesis screen in *Drosophila melanogaster* identified growth control phenotypes in the developing eye. One mutant line from this screen, *H.3.2*, was phenotypically characterized using the FLP/FRT system and genetically mapped by complementation analysis and genomic sequencing by undergraduate students participating in the multi-institution Fly-CURE consortium. *H.3.2 *was found to have a nonsense mutation in *short stop* (*shot*), an**ortholog of the mammalian spectraplakin *dystonin* (*DST*). *shot *and *DST* are involved in cytoskeletal organization and play roles during cell growth and proliferation.

**Figure 1. Characterization of shotH.3.2 mutation by phenotypic analysis, complementation mapping, and genetic sequencing:**
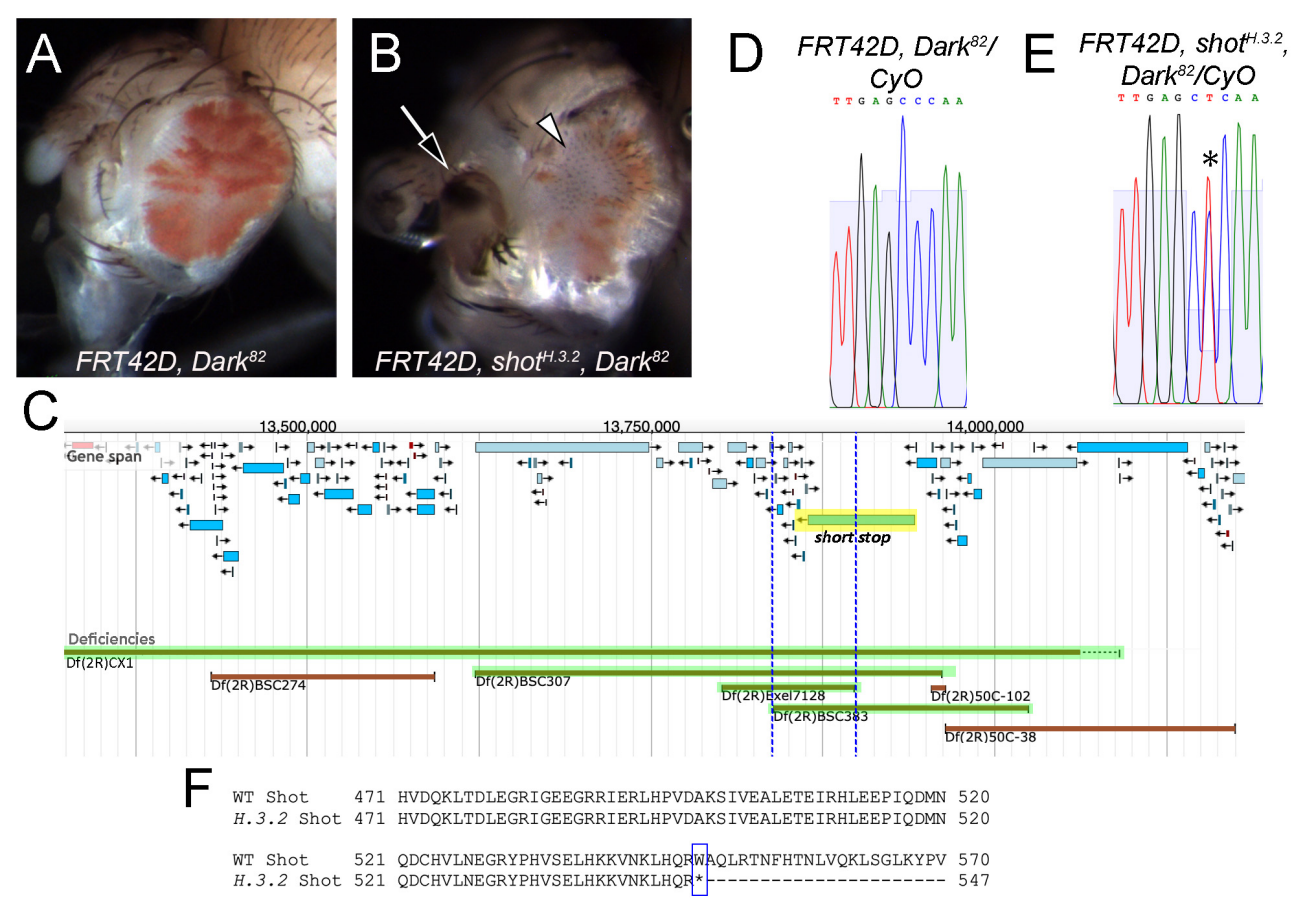
*FRT42D, Dark^82^* control mosaic eyes **(A)** exhibit an increased ratio of red (mutant) to white (wildtype) tissue compared to *FRT42D,* *shot^H.3.2^, Dark^82^*mosaic eyes **(B)**. Mosaic eyes with *shot^H.3.2^, Dark^82^* clones display antennal defects (arrow) and disorganized wildtype ommatidia (arrowhead). **(C)** Map of deficiency lines that complemented (red bars) and failed to complement (highlighted in green) the *H.3.2* mutation, resulting in a region of overlap of 2R:13,839,479..13,897,827 (between blue dashed lines). *short stop* (*shot*) is highlighted in yellow. Adapted from FlyBase using release FB2021_03 (Larkin *et al.* 2021). **(D-E)** The nucleotide location of *H.3.2* was identified by sequence analysis. *FRT42D, Dark^82^* control sequence with a single C peak at 2R:13,904,428 in the coding region of *shot* **(D)**, compared to *FRT42D, shot^H.3.2^, Dark^82^* mutant sequence with a heterozygous double peak revealing a nucleotide change from C to T at this position (**E**, asterisk). **(F)** Amino acid alignment of wildtype (WT) and *H.3.2* mutant Shot sequences. Amino acids 471 to 570 encode a spectrin-like repeat in Shot isoform H. The Trp548* mutation in the *H.3.2* mutant allele is indicated (box).

## Description

The homozygous lethal *H.3.2* mutation was generated from an EMS mutagenesis screen utilizing the FLP/FRT mitotic recombination system on chromosome 2R to detect phenotypic abnormalities consistent with aberrant cell growth control in the developing eye (Kagey *et al.* 2012). Apoptosis was prevented using an allele of *Death-associated Apaf-related killer* (*Dark*;allele *Dark^82^*), which carries a *mini-white* transgene (*w^+mC^*) and allows for mutant cells to be identified by the presence of red pigmentation after mitotic recombination (Akdemir *et al.* 2006).

The phenotypic analysis and genetic mapping of mutant *H.3.2* were carried out by independent groups of undergraduate researchers in genetics laboratory courses at three different institutions as part of the Fly-CURE consortium (Bieser *et al.* 2018). First, phenotypic analysis of *H.3.2* was performed by crossing flies expressing the site-specific recombinase FLP under the control of a promoter for *eyeless* (*ey*) and FLP target sites on chromosome 2R (*FRT42D*) with flies carrying the *H.3.2* mutation (genotype *FRT42D, H.3.2, Dark^82^/CyO*) or control flies (genotype *FRT42D, Dark^82^/CyO*). Mosaic eyes of the resulting progeny were quantified, showing that the *H.3.2* mutation resulted in mosaic eyes with less mutant tissue (17% on average; Figure 1B) than control eyes (49% on average; Figure 1A). In addition, disorganized patterning of the ommatidia resulting in a rough eye phenotype, antennal defects, and other morphological defects in the tissue surrounding the eye were observed in *H.3.2* mutant eyes (Figure 1B), but not in control eyes (Figure 1A). These data suggest that *shot* may be a conditional suppressor of proliferation non-cell autonomously and/or may promote proliferation cell autonomously.

Genetic complementation mapping of the *H.3.2* mutation was performed by crossing *FRT42D, H.3.2, Dark^82^/CyO* females with males from each of the stocks in the Bloomington 2R Deficiency Kit (Cook *et al.* 2012). At least fifty progeny from each cross were evaluated for complementation based on lethality or survival of the homozygous lethal *H.3.2* mutation in trans to each deficiency chromosome. Chromosomal breakpoints were obtained from the Bloomington *Drosophila* Stock Center for further analysis (Table 1). *H.3.2* failed to complement deficiency lines *Df(2R)CX1/SM1*, *Df(2R)BSC307/CyO*, and *Df(2R)BSC383/CyO* (Table 1, Figure 1C). Additional crosses were performed with smaller deficiencies within the region (Table 1), allowing for a more refined region of overlap to 2R:13,839,479..13,897,827 (Figure 1C). Alleles of the gene *short stop* (*shot*), one of the 12 protein-coding genes in the region, failed to complement the *H.3.2* mutation (Table 1).

**Table 1.** Complementation results with 2R deficiency lines and individual alleles of *shot* with *FRT42D, H.3.2, Dark^82^.* Deficiency mapping narrowed down the region of the *H.3.2* mutation to 2R:13,839,479..13,897,827, which contains the gene *short stop* (*shot*).

**Table:** 

**Bloomington 2R Deficiency Kit**			
**Deficiency**	**BDSC Stock #**	**Region**	**Complementation Result**
*Df(2R)CX1/SM1*	442	2R:12,700,421..14,091,140	Fail to complement
*Df(2R)BSC274/CyO*	23170	2R:13,430,464..13,593,272	Complement
*Df(2R)BSC307/CyO*	23690	2R:13,623,008..13,961,601	Fail to complement
*Df(2R)BSC383/CyO*	24407	2R:13,839,479..14,024,879	Fail to complement
*Df(2R)50C-38/CyO*	8114	2R:13,964,325..14,175,325	Complement
**Additional Deficiency Lines**			
**Deficiency**	**BDSC Stock #**	**Region**	**Complementation Result**
*Df(2R)Exel7128/CyO*	7873	2R:13,801,956..13,897,827	Fail to complement
*Df(2R)50C-102/CyO*	8111	2R:13,954,125..13,964,325	Complement
**Individual Gene Alleles**			
**Genotype**	**BDSC Stock #**	**Gene of interest**	**Complementation Result**
*b,^1^ wb^SF20^, Adh^n4^, shot^SF20^/CyO*	29033	*shot*	Fail to complement
*FRT42D, shot^V104^/SM5*	8740	*shot*	Fail to complement

Because complementation mapping indicated candidate gene *shot* as a potential location for the *H.3.2* mutation, genomic DNA was isolated from *FRT42D, H.3.2, Dark^82^/CyO* mutant and *FRT42D, Dark^82^/CyO* control flies for PCR and sequence analysis. Primers were designed to amplify exons of the *shot* gene and obtained from Integrated DNA Technologies. PCR products were purified and sequenced by Eurofins Genomics, Inc. to determine the molecular nature of the *H.3.2* mutation.

Sequence analysis revealed a nucleotide change at 2R:13,904,428, corresponding to *shot*, in *H.3.2* heterozygous flies. Where the control sequence showed a single C peak at this position (Figure 1D), the *H.3.2* mutant sequence showed two peaks of T and C (Figure 1E). This change results in a nonsense mutation, wherein a tryptophan is mutated to a premature stop codon in all 21 of the annotated polypeptides encoded by the *shot* gene. This corresponds to a Trp548* change in a predicted spectrin-like repeat of isoform H, the longest annotated Shot polypeptide containing 8805 amino acids (Figure 1F). Therefore, this mutation results in Shot loss-of-function and the observed *H.3.2* mutant phenotype. The increase of wildtype (white) cells in *H.3.2* mutant mosaic tissue suggests a possible non-cell autonomous role of *shot* in eye development, wherein mutant cells signal to neighboring wildtype cells, promoting their proliferation. It is also possible that *shot* promotes cell proliferation autonomously (Su 2015). Additional experimentation is needed to better elucidate the molecular roles of *shot* during eye development.

Based on genetic mapping and sequence analysis in *Drosophila melanogaster*, we conclude that *H.3.2* is a novel mutant allele of *shot* (*shot^H.3.2^*), resulting in a truncated protein. *shot* encodes a spectraplakin that interacts with microtubules, scaffold proteins, and certain signaling factors, and has been implicated in the development and maintenance of various tissues, including the nervous system, epidermis, and wing (Ricolo and Araujo 2020). *shot* was recently shown to have a role in cell growth via control of mitotic spindle assembly and chromosome dynamics (Dewey *et al.* 2020). RNAi knockdown of *shot* in *Drosophila* epithelia results in DNA double-stranded breaks (DSBs) due to errors in chromosome segregation during mitosis. *shot* has also been shown to play a role in regulating certain signaling molecules that direct cell spreading by controlling the actin cytoskeleton, thereby indicating a potential role for *shot* in suppressing tumor growth (Jain *et al.* 2019). Disruption of these or other mechanisms may contribute to the *H.3.2* mutant phenotype observed in the eye.

The mammalian ortholog of *shot*, *dystonin* (*DST*), encodes an adhesion junction protein active in epithelial, neuronal, and muscular tissue, and is implicated in cell adhesion, cytoskeleton organization, and cell migration (Künzli *et al.* 2016). In addition, while *shot* was recently shown to suppress the transcriptional regulator YAP via mechanical cues, *DST* was similarly shown to be a novel tumor suppressor in the Hippo pathway (Jain *et al.* 2019). Therefore, future characterization of *shot^H.3.2^* in *Drosophila* may contribute to evidence linking the cytoskeletal functions of these genes to growth control via cell autonomous and non-cell autonomous mechanisms.

## Reagents

*w^–^; FRT42D, Dark^82^/CyO* (Akdemir *et al*. 2006)

*w^–^; FRT42D, shot^H.3.2^, Dark^82^/CyO* (this study)

*w^–^, ey-FLP; FRT42D* (BDSC 5616)

Bloomington *Drosophila* Stock Center 2R Deficiency Kit (Cook *et al.* 2012)

*Df(2R)Exel7128/CyO* (BDSC 7873)

*Df(2R)50C-102/CyO* (BDSC 8111)

*b^1^, wb^SF20^, Adh^n4^, shot^SF20^/CyO* (BDSC 29033)

*FRT42D, shot^V104^/SM5* (BDSC 8740)

*shot* forward primer: 5’ CCAACTTGTTTTGGCACCACTC 3’

*shot* reverse primer: 5’ CGAGGTTATCCTTCAGCAGG 3’
